# Negative Video Head Impulse Test in Acute Vestibular Syndrome Does Not Exclude Vestibular Neuritis: Insights and Challenges in Diagnosis

**DOI:** 10.1002/ccr3.70300

**Published:** 2025-03-16

**Authors:** Pavol Skacik, Stefan Sivak, Egon Kurca

**Affiliations:** ^1^ Neurology Department University Hospital Martin Martin Slovakia; ^2^ Jessenius Faculty of Medicine in Martin Comenius University Bratislava Bratislava Slovakia

**Keywords:** acute vestibular syndrome, vertigo, vestibular neuritis, vestibulo‐ocular reflex, video head impulse test

## Abstract

Video Head Impulse Test (v‐HIT) is a valuable tool for diagnosing acute and chronic vestibular disorders but may yield false negatives in acute vestibular neuritis. Clinical judgment remains paramount; integrating patient history, physical findings, and ancillary tests ensures accurate diagnosis, especially when v‐HIT results conflict with the clinical picture and other diagnostic tools.

## 1 Introduction

Acute vestibular syndrome (AVS) is characterized by the sudden onset of vertigo, dizziness, or imbalance, often accompanied by findings such as nystagmus, gait unsteadiness, and nausea or vomiting [1]. In the acute stage, the clinician must address the critical question: “Is it central or peripheral?” This distinction is crucial given the potential for life‐threatening etiologies, such as stroke. The primary diagnoses responsible for spontaneous AVS include vestibular neuritis (or acute unilateral vestibular neuropathy), Ménière's disease, pseudo‐vestibular neuritis due to brainstem lesions, and vestibular migraine.

In recent years, significant advancements have been made in ancillary vestibular diagnostic tests, with one test—v‐HIT—gaining particular popularity due to its ease of use. This test assesses the function of the semicircular canals in all three planes of stimulation—roll, yaw, and pitch—covering all six canals. It provides clinicians with valuable insight into semicircular canal function, making it useful for evaluating both acute and chronic vestibular disorders.

In 2015, Newman‐Toker et al. proposed that v‐HIT helps address a key question in the emergency room: a normal vestibulo‐ocular reflex (VOR) observed during v‐HIT is more likely to indicate a central origin, such as a stroke. Incorporating v‐HIT into the well‐known HINTS criteria (Head Impulse, Nystagmus, Test of Skew) can increase diagnostic accuracy when evaluating patients with acute vertigo and dizziness, aiding in the differentiation between peripheral, central, and nonvestibular causes of vertigo [2].

Despite its growing use, v‐HIT may yield false negatives in some cases of vestibular neuritis, posing diagnostic challenges. The findings of this case‐based study underscore the importance of integrating v‐HIT with clinical evaluation, imaging, and patient history to ensure an accurate diagnosis in AVS.

### 1.1 Case Presentation 1

The case describes an atypical clinical presentation in a 24‐year‐old male who presented to the emergency department with dizziness, unsteadiness, and worsening balance that he first noticed upon waking. He was not taking any medications and had no prior diagnoses, nor did he report any recent signs of infection.

### 1.2 Diferential Diagnosis 1

During clinical examination, the patient exhibited no nystagmus, and the HINTS‐plus criteria (bedside HIT, nystagmus, test of skew, and hearing) were negative. He showed no signs of motor, sensory, or brainstem dysfunction. Upon evaluating his stance and gait, his stance was slightly wide‐based with paleocerebellar characteristics. The Romberg test was positive with truncal sway; however, there were no signs of vestibulospinal asymmetry. Basic laboratory tests were normal, and both CT and CT angiography revealed no pathological findings. Audiometry results were within normal limits.

**FIGURE 1:**
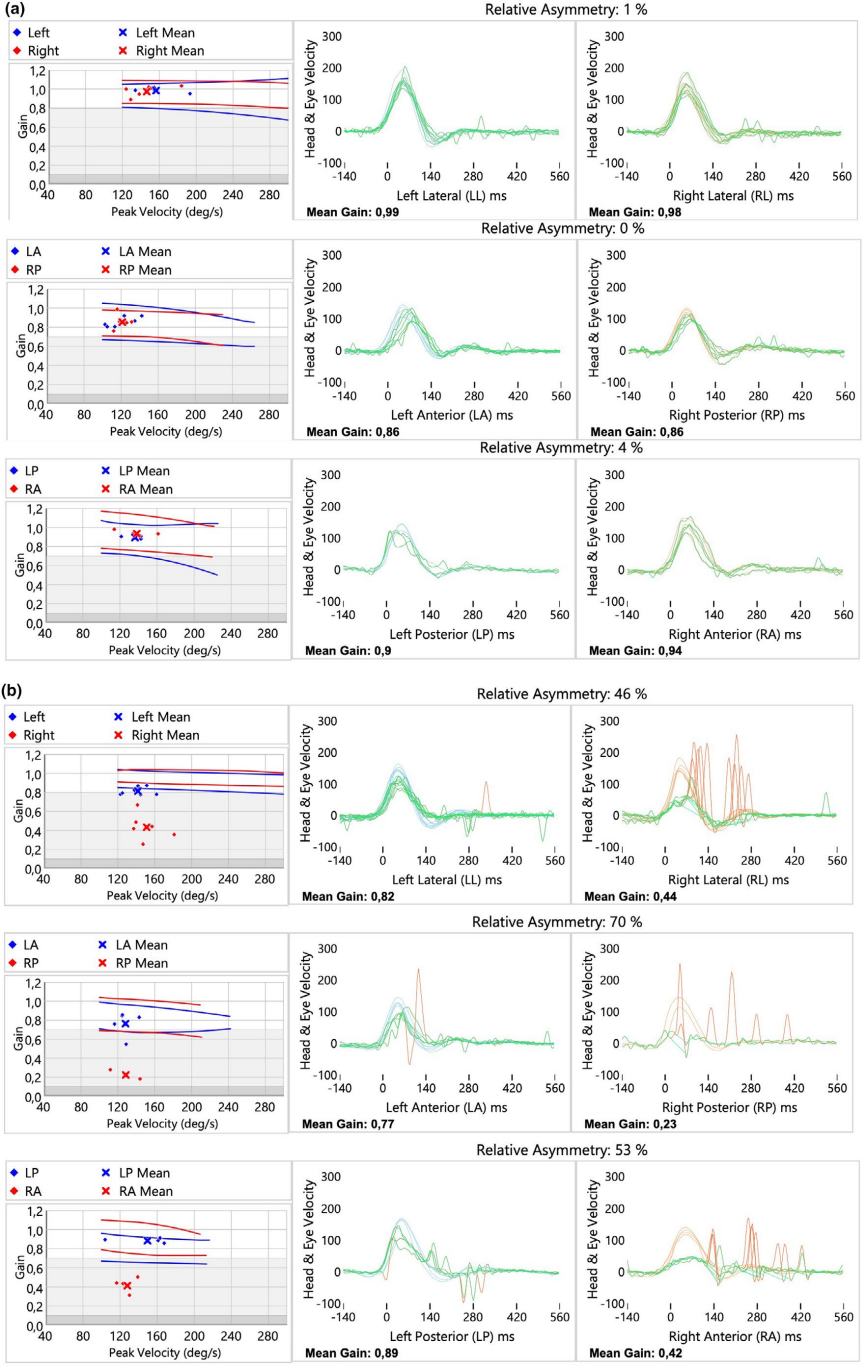
(a, b) V‐HIT test in the acute stage. The patient did not show any pathological signs, with physiological gain values and no corrective saccades (a) V‐HIT test performed more than 72 h after the onset of symptoms indicated total unilateral vestibular loss on the right side, with reduced gain values and the presence of corrective covert and overt saccades indicating complete vestibular nerve lesion (b), Fewer trials for the right posterior canal may affect result reliability. Poor head and neck positioning tolerance affected the testing v‐HIT of the anterior and posterior semicircular canals.

On Day 1 of admission, the patient underwent a v‐HIT; the results, shown in Figure 1, indicated physiological findings for the lateral semicircular canals (the examination was performed within approximately 8 h of symptom onset). Because of these findings, there was clinical suspicion of central vestibular syndrome. The patient then underwent a 3‐Tesla MRI of the brain, which revealed no pathological changes in signal. On Day 4 of hospitalization, the patient was clinically stable, with no changes in his neurological status. A repeat v‐HIT was performed, which now showed reduced gain and numerous covert and overt saccades (as shown in Figure 1). A diagnosis of vestibular neuritis was made.

### 1.3 Conclusion and Results 1

The patient was managed with pharmacological symptomatic treatment and initiated vestibular rehabilitation during his hospitalization. He experienced a complete resolution of symptoms within 2 weeks after discharge.

### 1.4 Case Presentation 2

A 66‐year‐old female was admitted to the Neurology Department due to spontaneous, persistent vertigo. Her vertigo was aggravated by changes in position and exacerbated by visual stimuli, such as moving objects and scrolling on her mobile phone. Her medical history included treatment for grade I arterial hypertension (ESC/ESH) and dyslipidemia. On admission, an ECG revealed paroxysmal atrial fibrillation.

### 1.5 Differential Diagnosis 2

According to the HINTS‐plus criteria, the clinical picture was negative, although there was a slight disruption in smooth pursuit movements in both the horizontal and vertical planes. The patient exhibited asymmetry of vestibulospinal reflexes; during the Unterberg test, there was significant deviation to the left. Nystagmography revealed no spontaneous or gaze‐evoked nystagmus. Induced head‐shaking nystagmus, recorded via videonystagmography, showed horizontal nystagmus with the fast phase beating to the right for approximately 5 to 7 beats without any change in direction.

**FIGURE 2:**
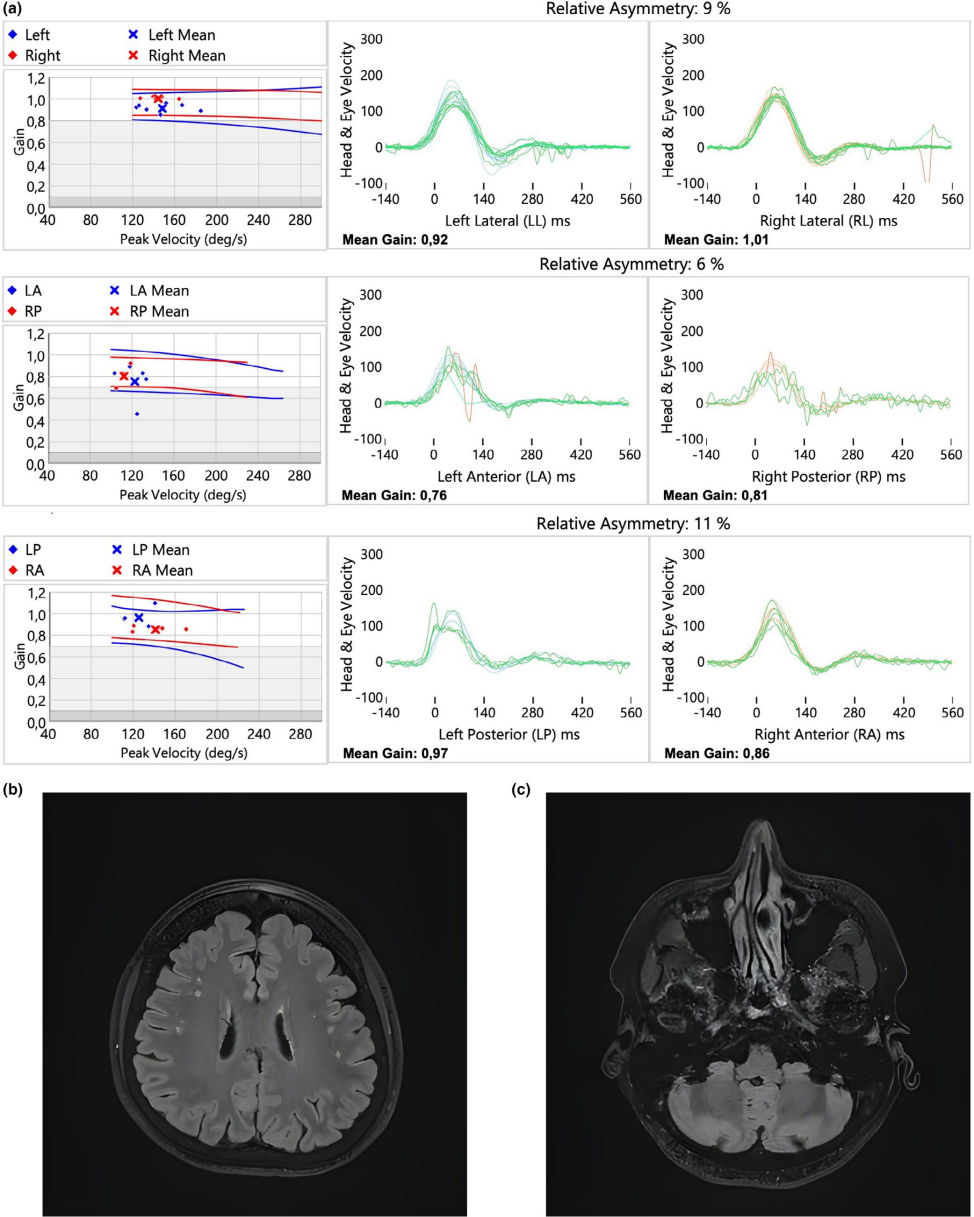
(a–c) V‐HIT test during the acute stage of a patient with mixed clinical and ancillary test results. We observed physiological gain values and no significant corrective saccades (a) MRI, infratentorially, there were no pathological signal changes, supratentorially, bilateral vascular changes were noted, classified as vascular encephalopathy—Fazekas I (b, c).

Positional tests were positive. Both the Dix‐Hallpike maneuver (performed on both ears for the anterior and posterior semicircular canals) and the Pagnini‐McClure maneuver (performed on both ears for the lateral semicircular canals) evoked positional nystagmus with a latency of 0–2 s. The evoked nystagmus was vertical, up‐beating (toward the forehead), with a stable slow‐phase velocity (average SPV values of approximately 7°–10° per second) and persisted for more than 60s in all positions. Repositioning maneuvers did not alleviate the nystagmus, which was reproducible, although the patient did not report any subjective vertigo or dizziness during these maneuvers. The v‐HIT did not reveal any findings typically associated with a peripheral lesion (shown in Figure 2).

Due to discrepancies between the clinical and ancillary tests, an MRI was performed. The MRI revealed no pathological changes in the vestibular nerve, brainstem, or cerebellum, nor were any acute signal changes observed in other brain regions. Supratentorially, bilateral hyperintense lesions of vascular origin were noted, classified as Fazekas I (shown in Figure 2). Audiometry showed normal results, and both CT of the brain and CT angiography were unremarkable.

### 1.6 Conclusion and Results 2

Regarding the suspected peripheral vestibular lesion, the patient was treated with a combination of antivertiginous drugs for the first 3 days, corticosteroids during the acute stage, and vestibular rehabilitation. Given her vascular risk factors (hypertension, dyslipidemia, and paroxysmal atrial fibrillation), she was initiated on statin therapy, antihypertensive medications, and oral anticoagulation. Vestibular rehabilitation led to complete resolution of symptoms within 2–3 weeks.

## 2 Discussion

The Head Impulse Test, introduced in 1988, evaluates the VOR to assess canal paresis in vestibular disorders [3]. However, it has two main drawbacks: examiners may miss corrective saccades, and its accuracy depends on subjective evaluation. In 2009, the v‐HIT emerged as a solution, quantifying the severity of VOR impairment [4]. Halmagyi et al. noted that even minor VOR impairments can be detected using v‐HIT [5]. As mentioned, v‐HIT is now increasingly used to diagnose both acute and chronic vestibular disorders [6].

For vestibular neuritis, diagnostic criteria require clear evidence of reduced VOR function on the side opposite the fast phase of spontaneous nystagmus [6]. Although some studies report pathological v‐HIT results in 91%–98% of patients with acute vestibular neuritis [7, 8, 9, 10, 11, 12], findings are inconsistent. For instance, Lee et al. observed normal v‐HIT results in up to 81% of such patients [13], suggesting that unequivocal evidence of VOR dysfunction may not always be present.

In the first patient, vestibular neuritis presented as a dynamic process with heterogeneous and atypical clinical features. In the hyperacute stage, v‐HIT findings were normal, which could lead to an incorrect diagnosis. This case emphasizes the potential value of repeating v‐HIT evaluations during the first days of the disease. Another issue in vestibular neuritis is the discrepancy between clinical findings and ancillary test results. While the clinical picture—such as asymmetry in vestibulo‐spinal reflexes and corresponding head‐shaking nystagmus—suggests a peripheral etiology, both bedside tests and v‐HIT can sometimes be negative. In such cases, caloric testing is recommended. Although v‐HIT is easier and less time‐consuming than caloric testing, the two assess different frequency ranges: caloric testing evaluates low frequency vestibular function (typically less than 0.005 Hz), whereas v‐HIT examines more physiological frequencies (1–5 Hz). Caloric testing is generally considered more sensitive for detecting vestibular dysfunction. For example, one study found that only 33% (17/51) of patients with unilateral vestibular dysfunction had abnormal v‐HIT results, and among these, most had abnormal caloric responses [14]. Perez et al. reported that abnormal v‐HIT results appeared when unilateral weakness reached 42.5% on caloric testing, yet the reason for the dissociation between these tests in acute vestibular neuritis remains unclear [15]. A comparison of the advantages and limitations of diagnostic tests is described in Table 1.

**TABLE 1:** Comparision of diagnostic value of bedside HIT, v‐HIT and caloric testing in patients with vestibular neuritis.

Test	Advantages	Limitations
Bedside HIT	Can be performed quickly at the patient's bedside Requires no specialized equipment Moderate sensitivity (variable results) and high specificity in expert hands	Generally has low sensitivity and specificity It is a nonquantitative, subjective test that depends heavily on examiner expertise
v‐HIT	Quantitative measure of the VOR gain High diagnostic value Higher sensitivity and specificity	Requires specialized equipment Technical expertise needed for accurate interpretation Results may vary depending on the equipment and analysis system used
Caloric testing	Well‐established method. Quantitative assessment of unilateral vestibular function	Requires specialized equipment Time‐consuming May be uncomfortable for patients Limited to low frequency responses Stimulating mainly lateral semicircular canals

A second case revealed a discrepancy between the clinical presentation and ancillary tests. The patient exhibited acute vestibular syndrome alongside positional nystagmus and impaired smooth pursuit on videonystagmography. These findings might result from vascular supratentorial lesions—where central positional nystagmus without vertigo represents a central positional vestibular syndrome—or from diffuse cerebral lesions causing nonspecific smooth pursuit dysfunction. Alternatively, a peripheral acute vestibular syndrome with negative v‐HIT results could indicate other etiologies as the first attack of vestibular Ménière's disease or vestibular migraine. It is also possible that an acute central lesion (stroke) was missed on MRI or that a sudden interruption of blood flow in the anterior vestibular artery mimicked superior vestibular nerve inflammation.

Management in such cases must be comprehensive. For the suspected peripheral vestibular lesion, treatment included antivertiginous drugs during the first 3 days, corticosteroids in the acute stage, and vestibular rehabilitation. Given the patient's vascular risk factors (hypertension, dyslipidemia, and paroxysmal atrial fibrillation), therapy also included statins, antihypertensives, and oral anticoagulation.

The exact mechanism behind negative v‐HIT results in the acute stages of vestibular neuritis remains under discussion. Several explanations have been proposed. For example, v‐HIT assesses a limited frequency range, while other tests evaluate different ranges. This suggests that different vestibular sensory cells might be affected—particularly since type II cells, which do not respond to high‐frequency stimuli and may remain intact. Additionally, a partial or incomplete lesion of the vestibular nerve may damage only a subset of fibers—potentially fewer than the threshold detectable by v‐HIT. Central compensatory mechanisms may also be in effect during v‐HIT testing. The cerebellum plays a key role in modulating vestibular nuclei activity, recalibrating neuron sensitivity, and reducing symptoms rapidly. These compensatory processes can vary among patients based on factors such as age‐related declines in neuronal plasticity, the extent of vestibular damage, coexisting visual or somatosensory deficits (e.g., cataracts, polyneuropathy), participation in vestibular rehabilitation, prolonged use of vestibular suppressants, psychological factors, and previous cerebellar dysfunction. Furthermore, the timing of v‐HIT testing is crucial, as compensatory mechanisms may already mask underlying deficits [4]. Technical challenges such as goggle slippage, calibration issues, operator inexperience, and patient‐related factors can also lead to inaccurate v‐HIT results [16, 17, 18].

Vestibular neuritis is a dynamic condition with variable courses—from mild, episodic symptoms to prolonged recovery. Although the study provides valuable insights into negative v‐HIT results, it has methodological and technical limitations. Future research should use comprehensive testing, standardized protocols, larger samples, and long‐term follow‐up to improve reliability.

## 3 Conclusion

Recent publications on v‐HIT highlight its high sensitivity and specificity for diagnosing vestibular neuritis and its negative predictive value for central pathology. However, patients with peripheral vestibular syndrome may still have negative v‐HIT results. This discrepancy could be due to partial vestibular lesions, loss of fibers linked to high‐frequency stimulation, or the timing of the test when compensatory mechanisms have begun.

Ultimately, the clinical picture is paramount. Vestibular neuritis is primarily a clinical diagnosis and cannot be ruled out based solely on a normal v‐HIT. When a patient exhibits typical vestibular nystagmus, can stand and walk, shows vestibulo‐spinal asymmetry consistent with the nystagmus, and lacks brainstem, motor, sensory, cerebellar, or hearing symptoms—even with a normal bedside or v‐HIT—the etiology is likely peripheral. Clinicians should integrate the overall clinical presentation, patient history, comorbidities, and ancillary test or imaging findings.

## Author Contributions

**Pavol Skacik:** conceptualization, investigation, methodology, writing – original draft, writing – review and editing. **Stefan Sivak:** supervision, validation, writing – review and editing. **Egon Kurca:** writing – review and editing.

## Consent

Written informed consent was obtained from all the patients to publish this report in accordance with the journal's patient consent policy.

## Conflicts of Interest

The authors declare no conflicts of interest.

## Data Availability

The data supporting the findings of this study are available from the corresponding author upon reasonable request.
